# Optimal Planting Density Increases the Seed Yield by Improving Biomass Accumulation and Regulating the Canopy Structure in Rapeseed

**DOI:** 10.3390/plants13141986

**Published:** 2024-07-20

**Authors:** Guobing Lin, Long Wang, Yiyang Li, Jing Li, Chen Qian, Xia Zhang, Qingsong Zuo

**Affiliations:** 1 Jiangsu Key Laboratory of Crop Genetics and Physiology, Yangzhou University, Yangzhou 225009, China; mx120220714@stu.yzu.edu.cn (G.L.); dx120210110@yzu.edu.cn (L.W.); mx120230775@stu.yzu.edu.cn (Y.L.); mx120210720@yzu.edu.cn (J.L.); mz120211279@yzu.edu.cn (C.Q.); 2 Jingyuan Rapeseed Production Professional Cooperative, Jiangdu, Yangzhou 225009, China; zhangxia202407@163.com; 3 Jiangsu Co-Innovation Center for Modern Production Technology of Grain Crops, Yangzhou University, Yangzhou 225009, China

**Keywords:** planting density, yield, biomass accumulation and partitioning, canopy, light interception and distribution

## Abstract

Planting density is an important factor affecting plant growth and yield formation in rapeseed. However, the understanding of the mechanism underlying the impact of planting density on biomass, canopy, and ultimate seed yield remains limited. A field experiment was conducted to investigate the effect of planting density on seed yield, yield components, biomass accumulation and partitioning, and canopy structure. Five planting density levels were set as D1 (2.4 × 10^5^ plants ha^−1^), D2 (3.6 × 10^5^ plants ha^−1^), D3 (5.4 × 10^5^ plants ha^−1^), D4 (6.0 × 10^5^ plants ha^−1^), and D5 (7.2 × 10^5^ plants ha^−1^). The results showed that with planting density increasing from D1 to D3, the seed yield, number of pods in population, and 1000-seed weight increased, while seedling survival rate, yield per plant, number of pods per plant, and number of seeds per plant decreased. When planting density increased to D4 and D5, seed yield dramatically decreased due to a decreased number of seeds per pod and 1000-seed weight. Increasing planting density from D1 to D3 increased biomass accumulation in all organs. D3 produced the highest biomass partitioning in seeds. In addition, D2 and D3 treatments had a high level of pod area index (5.3–5.8), which caused an approximately 93% of the light to be intercepted. The distribution of light in D2 and D3 was more evenly spread, with the upper and lower parts of the canopy displaying a distribution ratio of roughly 7:3. Therefore, D2 and D3 produced the highest seed yields. In conclusion, D2 and D3 are recommended in rapeseed production due to their role in improving biomass accumulation and partitioning and canopy structure.

## 1. Introduction

Rapeseed is an important oilseed crop, with its seed serving not only edible oil production but also as feedstock for biofuel manufacturing and biodiesel synthesis in the chemical industry [1]. Rapeseed is cultivated globally, and China stands out as one of the leading countries cultivating this crop, with a planting area of around 7.25 million hectares in 2022 [2]. In recent years, with continuous advancements in cultivation techniques and comprehensive utilization of improved varieties, China has witnessed a steady increase in planting area and seed yield of rapeseed [3]. Given the challenges posed by a growing population and limited resource availability, enhancing rapeseed productivity to meet growing demands has become a prominent topic within rapeseed production.

The seed yield of rapeseed is determined by multiple factors, including the number of pods per unit area, number of seeds per pod, and 1000-seed weight. Adjusting cultivation practices can alter yield components and improve yield. For instance, increasing nitrogen fertilizer application could enhance both the number of pods in the population and seeds per pod, resulting in a higher seed yield [4,5]. Planting density is one of the most critical field management practices in rapeseed cultivation. A previous study demonstrated that increasing planting density could increase the number of pods in the population and achieve a greater seed yield [6]. However, an excessive density may deteriorate the population environment, such as lodging, shadowing, and increasing temperature, which would reduce the pod quality (number of seeds per pod and 1000-seed weight) [7]. The number of seeds per pod and 1000-seed weight are also important factors affecting seed yield. Generally, they are closely related to variety, although cultivation practices also have effects on them. The optimal planting density can effectively maintain a high seed number per pod and 1000-seed weight, while ensuring an adequate quantity of pods [8]. However, an excessive planting density would intensify individual competition, inhibiting pod growth and seed filling, resulting in a reduction in the number of seeds per pod and 1000-seed weight [9]. Therefore, it is crucial to determine an appropriate planting density to coordinate different yield components in rapeseed production.

Producing adequate biomass through cultivation practice management is a crucial basis for plant growth and seed yield [10]. The previous studies showed that increasing planting density could enhance biomass accumulation in the population and therefore improve grain yield in rice [11], wheat [12], maize [13], and rapeseed [14]. However, several studies reported that an excessive planting density negatively affected biomass accumulation, indicating that increasing biomass through increasing the planting density had a certain range [15]. Increasing the distribution of biomass in seed (harvest index) without increasing fertilizer input is conducive to promoting resource utilization efficiency and achieving green production. A study in cotton demonstrated that increasing the planting density could enhance biomass accumulation while decreasing the allocation to grains [16]. However, a study in maize showed that a moderate planting density enhanced nutrient transfer to grain and increased the harvest index while a high planting density decreased it [17]. Therefore, the response of biomass partitioning patterns depended on the specific plants and specific planting density. It is important to expand our knowledge of the effects of the planting density on biomass distribution patterns in rapeseed.

The canopy structure of rapeseed comprises main stems, branches, and pods, which play essential roles in physiological processes such as light utilization, nutrient absorption, and gas exchange [18,19]. Numerous studies have demonstrated that the canopy structure of rapeseed varies with different planting densities and that the quality of the canopy structure is closely related to yield formation [11,20]. During the seed-filling stage, pods in the canopy serve as primary photosynthetic organs [21]. An appropriate canopy structure can enhance light interception and improve light utilization efficiency [22,23]. Furthermore, an effective canopy structure not only effectively regulates the internal environment but also mitigates extensive lodging events [24,25]. Therefore, a favorable canopy structure under the optimal planting density is key for achieving a high yield in rapeseed.

In previous studies on the response of seed yield to the canopy structure, there was a lack of systematic integration of internal changes within the canopy. It is crucial to gain a deeper understanding of the effects of internal canopy variation on seed yield. Rapeseed plants have raceme inflorescence, with main raceme and branch racemes. The pods from both main raceme and branch racemes collectively contribute to the seed yield. A field experiment was conducted to investigate the effects of planting density on seed yield, yield components, and canopy structure throughout the growth process. We postulated that regulation of planting density can enhance the seed yield through the canopy structure.

## 2. Results

### 2.1. Plot Seed Yield

**Figure 1:**
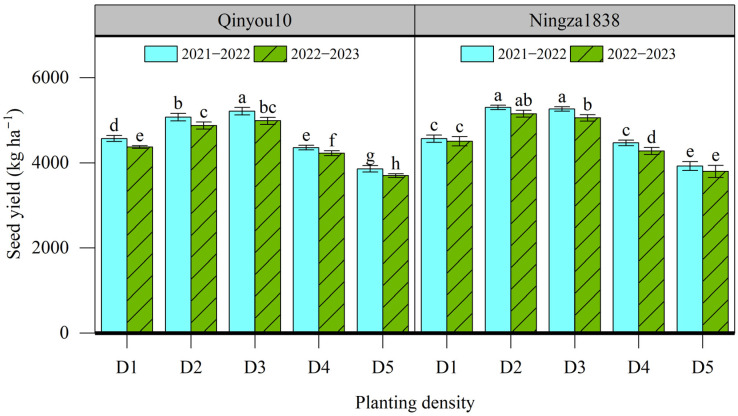
Effects of planting density on seed yield of canola plants. Different letters indicate significant difference at *p* = 0.05 between different planting density levels within same varieties. D1, 2.4 × 10^5^ plants ha^−1^; D2, 3.6 × 10^5^ plants ha^−1^; D3, 5.4 × 10^5^ plants ha^−1^; D4, 6.0 × 10^5^ plants ha^−1^; D5, 7.2 × 10^5^ plants ha^−1^.

The results of the plot seed yield at different planting density levels are presented in Figure 1. The seed yield for Qinyou10 ranged from 3700 to 5215 kg ha^−1^, while for Ningza1838, it ranged from 3799 to 5303 kg ha^−1^. The seed yield of two varieties in the 2021–2022 growing season was slightly higher than those in the 2022–2023 growing season. The seed yield for two varieties in two growing seasons showed similar tendencies in response to planting density. The seed yield of Qinyou10 and Ningza1838 increased by 14.1% and 15.2% in the 2021–2022 growing season, and by 14.1% and 12.2% in the 2022–2023 growing season, respectively, as the planting density increased from D1 to D3. Once the planting density exceeded D3, the seed yield decreased with increasing density. The lowest seed yield was recorded in D5.

### 2.2. Yield Components

The ANOVA results revealed that both variety and planting density had a significant effect on yield components, as indicated in Table 1. The seedling survival rate ranged from 89.0% to 97.9%, showing a decreasing tendency in response to an increase in planting density. Similarly, as the planting density increased from D1 to D5, there was a significant decrease in the number of pods per plant, number of seeds per pod, and yield per plant. For Qinyou10, the decreases were 58.6%, 14.4%, and 69.5%, respectively. For Ningza1838, the declines were 57.9%, 14.5%, and 69.4%, respectively. The average 1000-seed weight for Ningza1838 was 3.53 g, which was greater than that of Qinyou10 (3.40 g). An increasing trend was observed in the 1000-seed weight of two varieties as the planting density increased from D1 to D3. When the planting density exceeded D3, the 1000-seed weight significantly decreased. Moreover, the number of pods in the population increased as the planting density increased, reaching the highest value at D4. Compared to D4, the number of pods in the population in D5 decreased, although it remained greater than in D2.

### 2.3. Biomass Accumulation and Partitioning

The biomass accumulation under different planting densities is shown in Table 2. A slightly higher biomass accumulation in the 2021–2022 growing season can be seen than in the 2022–2023 growing season. The biomass under different density levels ranged from 1351.3 to 1860.2 kg ha^−1^ for roots, 6537.4 to 8612.2 kg ha^−1^ for stems, 4353.7 to 5497.8 kg ha^−1^ for pods, 4079.8 to 5529.3 kg ha^−1^ for seeds, and 16,322.7 to 21,399.3 kg ha^−1^ for the total plant, respectively. The biomass accumulation significantly increased as the planting density was raised from D1 to D3, with the greatest values recorded at D3. In comparison to D1, the biomass accumulation of the roots, stems, pods, seeds, and total plant in D3 exhibited rises of 4.2%, 8.4%, 12.6%, 13.3%, and 10.3%, respectively. Nevertheless, when the planting density surpassed D3, there was a significant drop in biomass across all organs. The lowest biomass was recorded in D5. In comparison to D3, the biomass accumulation of the roots, stems, pods, seeds, and total plant in D5 exhibited substantial decreases of 24.9%, 21.7%, 18.28%, 23.4%, and 21.6%, respectively.

The biomass partitioning in various organs under different planting densities is presented in Table 3. The biomass partitioning in the roots, varying between 8.25% and 9.20%, was lowest among different organs. The biomass was mainly located in the stems, accounting for 39.52% to 41.33%. With an increase in planting density, there was a noticeable decrease in the biomass partitioning in the roots and stems. In comparison to D1, the biomass partitioning in D2–D5 exhibited decreases of 4.7–9.6% for the roots and 1.0–2.0% for the stems, respectively. The biomass partitioning in the pods, which varied from 24.73% to 27.15%, was positively responsive to planting density. The biomass partitioning in the seeds increased as the planting density increased from D1 to D3. Compared to D1, the biomass partitioning in the seeds in D3 exhibited increases of 2.7% for Qinyou10 and 2.4% for Ningza1838, respectively. There was no significant difference in biomass partitioning in the seeds between D2 and D3. The biomass partitioning in the seeds in D4 and D5 was significantly lower than that in D2 and D3.

### 2.4. Yield Performance of Main Raceme and Branch Raceme

#### 2.4.1. Yield Components of Main Raceme and Branch Raceme

The yield components of main raceme and branch raceme during two growing seasons are presented in Table 4 and Table 5. The pod number in the main raceme across different planting densities ranged from 15.98 to 33.27 × 10^6^ ha^−1^, which was significantly higher than that in a single branch raceme (ranging from 3.15 to 12.66 × 10^6^ ha^−1^). An increase in planting density caused an increase in the pod number in the main raceme. With the branch raceme located from the top to the bottom of the plant, the pod number showed an initial increase followed by a subsequent decrease, reaching its peak in the intermediate branches. The increase in planting density resulted in a consistent upward trend in the number of pods on the branch raceme.

The seed number per pod ranged from 14.8 to 20.7 in D1, 12.5 to 18.9 in D2, 14.5 to 17.7 in D3, 8.5 to 18.1 in D4, and 10.6 to 16.1 in D5. As planting density increased, the seed number per pod of both main raceme and branch raceme decreased. There was no significant trend in the seed number per pod in different branch positions. However, the seed number per pod in the lower branch raceme was significantly lower than it in other positions.

The 1000-seed weight of the main raceme ranged from 3.284 to 4.089 g, which was higher than that of branch raceme (ranging from 2.724 to 3.935 g). The 1000-seed weight with branch raceme located from top to bottom in the plant showed a decreasing tendency. With the increasing planting density, the 1000-seed weight of both main raceme and branch raceme significantly decreased.

#### 2.4.2. Seed Yield of Main Raceme and Branch Raceme

**Figure 2:**
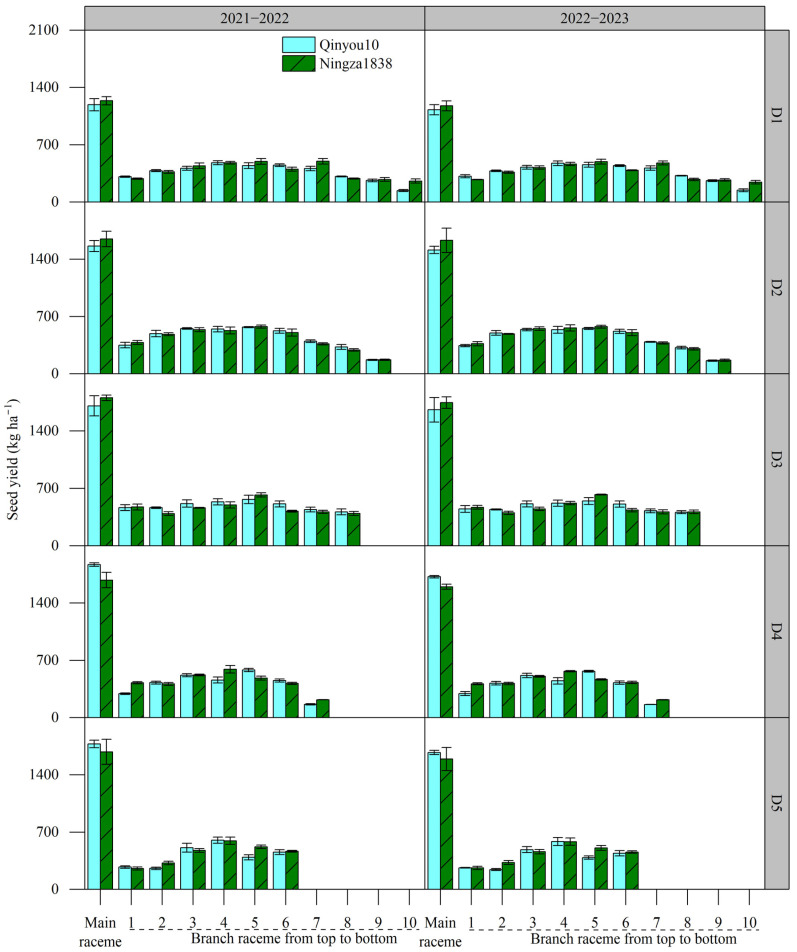
Effects of planting density on seed yield of main raceme and branch raceme. D1, 2.4 × 10^5^ plants ha^−1^; D2, 3.6 × 10^5^ plants ha^−1^; D3, 5.4 × 10^5^ plants ha^−1^; D4, 6.0 × 10^5^ plants ha^−1^; D5, 7.2 × 10^5^ plants ha^−1^.

The seed yield of main raceme and branch raceme under different planting density levels is shown in Figure 2. The seed yield of main raceme was significantly higher than that of single branch raceme. The seed yield of main raceme increased as the planting density increased. With the branches located from the top to the bottom of the plant, the seed yield initially increased and peaked at the intermediate branches, then decreased at the lower branches in the plant. However, the variation amplitude of the seed yield of branch raceme from the lowest value to the higher value varied across different planting density levels. The lowest variation amplitude was recorded at the rate of 44.5% in D3.

#### 2.4.3. Yield Distribution of Main Raceme and Branch Raceme

The yield distribution in main raceme and branch raceme is presented in Table 6. The yield distribution in main raceme ranged from 23.71% to 41.69% for Qinyou10 and 24.25% to 38.92% for Ningza1838, which was significantly higher than that in a single branch raceme. The distribution of yield in the main raceme was influenced by planting density, with a noticeable increasing trend as planting density increased. The distribution of yield varied among different branch racemes within the plant, depending on their respective locations. Generally, the yield distribution in the middle branch racemes was higher than those in the top and bottom branch racemes.

### 2.5. Canopy Structure

#### 2.5.1. PAI and Light Interception

**Figure 3:**
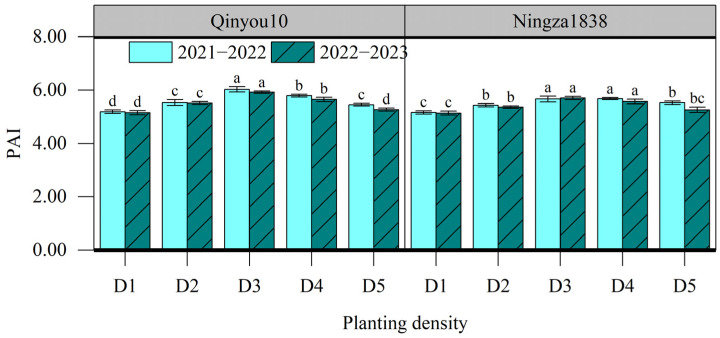
Effects of planting density on PAI at the seed-filling stage. D1, 2.4 × 10^5^ plants ha^−1^; D2, 3.6 × 10^5^ plants ha^−1^; D3, 5.4 × 10^5^ plants ha^−1^; D4, 6.0 × 10^5^ plants ha^−1^; D5, 7.2 × 10^5^ plants ha^−1^. Different letters indicate significant difference at *p* = 0.05 for the same year and same variety between different planting density levels.

PAI, ranging from 5.14 to 6.03, was significantly affected by planting density (Figure 3). As the planting density increased from D1 to D3, the PAI had a considerable rise of 12.9%. For Ningza1838, there was no significant difference in PAI between D3 and D4. However, there was a notable drop in PAI in D5 compared to D3. For Qinyou10, PAI exhibited a significant decrease when the planting density increased from D3 to D5.

**Figure 4:**
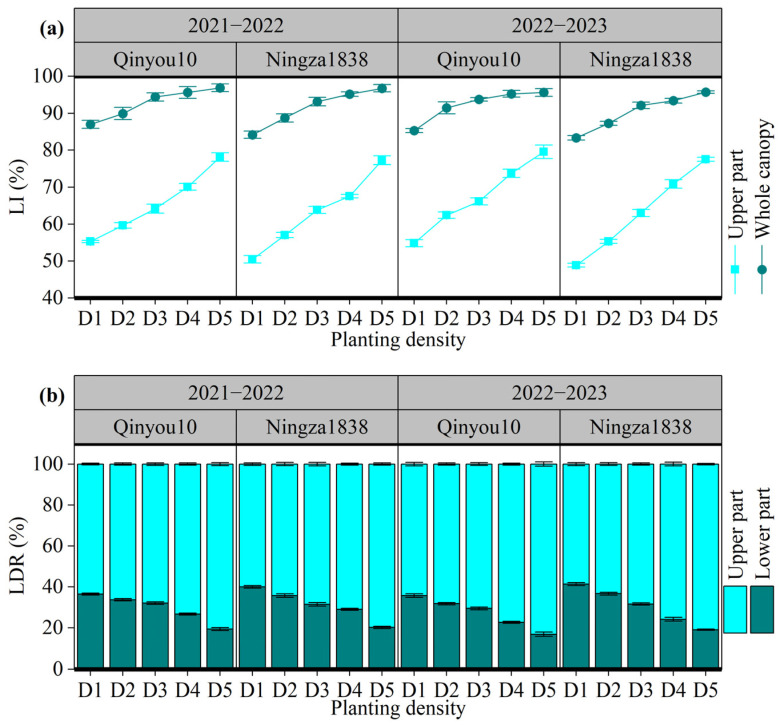
Effects of planting density on light interception and distribution at the seed-filling stage. (**a**): light interception (LI) of different parts of canopy. (**b**): light distribution ratio (LDR) of different parts of canopy. D1, 2.4 × 10^5^ plants ha^−1^; D2, 3.6 × 10^5^ plants ha^−1^; D3, 5.4 × 10^5^ plants ha^−1^; D4, 6.0 × 10^5^ plants ha^−1^; D5, 7.2 × 10^5^ plants ha^−1^. LI: light interception rate. LDR: light distribution ratio.

The light interception and distribution are presented in Figure 4. The LI ranged from 48.9% to 79.6% for the upper part of the canopy and from 83.3% to 96.9% for the whole canopy, as shown in Figure 4a. An increase in planting density resulted in a rise in LI in both the upper part of the canopy and whole canopy. However, there was a variation in the amplitude rise among different layers. For the upper part, the LI in D5 had a 49.2% rise compared to D1; for the whole canopy, the LI increased by 13.3%. Planting density also affected the light distribution among different parts (Figure 4b). Compared to the lower part, light was mainly distributed in the upper part. As planting density increased, LDR in the upper part increased, while LDR in the lower part decreased.

#### 2.5.2. Pod Photosynthesis

**Figure 5:**
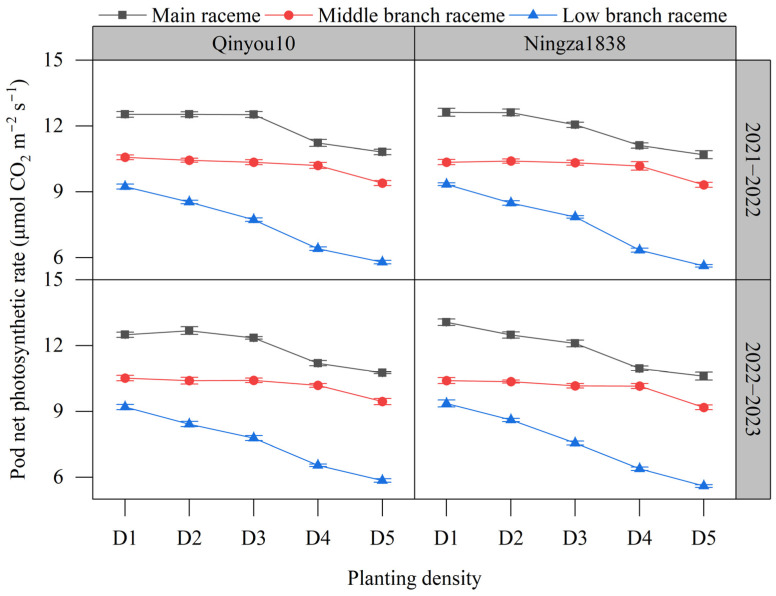
Effects of planting density on pod photosynthetic rate at the seed-filling stage. D1, 2.4 × 10^5^ plants ha^−1^; D2, 3.6 × 10^5^ plants ha^−1^; D3, 5.4 × 10^5^ plants ha^−1^; D4, 6.0 × 10^5^ plants ha^−1^; D5, 7.2 × 10^5^ plants ha^−1^.

The pod photosynthetic rate is presented in Figure 5. The order of pod photosynthetic rate among different positions was main raceme > middle branch raceme > low branch raceme. The pod photosynthetic rate decreased with the increasing planting density. Compared to D1, the pod photosynthetic rate of the main raceme in D5 declined by 15.4%. The pod photosynthetic rate of the middle branch raceme decreased by 10.8%. The pod photosynthetic rate of the low branch raceme decreased significantly more than the main stem and the middle layer, reaching 38.4%.

## 3. Discussion

### 3.1. Effects of Planting Density on Yield and Yield Components

Given the increasing demand for edible oil, it is important to improve rapeseed yield through cultivation adjustments. Planting density, as an agronomic practice, can effectively synchronize individual and population growth, thereby enhancing resource utilization and elevating crop yield. In our study, the seedling survival rate showed a consistent decline with increasing planting density, aligning with previous research [26]. This result may be due to increased competition among individuals resulting from higher density levels, consequently inhibiting pre-winter individual growth and increasing the likelihood of seedling death during the overwintering period [27,28]. Despite a decrease in seedling survival rate, the increase within a certain range in planting density still significantly influenced seed yield through population effects. The number of pods was identified as a crucial factor impacting seed yield. In this study, the number of pods per plant declined with increasing planting density, while the number of pods in the population increased and reached its peak at D4, which indicated that increasing planting density would inhibit individual plant growth due to reduced growing space for individuals while it could improve population growth [29]. The number of seeds per pod and 1000-seed weight are also significant factors influencing rapeseed seed yield [30,31]. In this study, both the number of seeds per pod and 1000-seed weight showed an overall decreasing trend with increasing planting density from D3 to D5. The increasing number of pods in the population caused reduced nutrient allocation for the individual pods, resulting in a decreased number of seeds per pod and 1000-seed weight [32]. In our study, the population pod reached a peak in D4 while D2 or D3 produced the greatest seed yield, which suggested that increasing seed yield through an increasing pod quantity had a certain range, and the planting density exceeding this range would deteriorate the canopy environment, resulting in dramatic decreases in the number of seeds per pod and 1000-seed weight, ultimately leading to substantial declines in seed yield [33,34]. In conclusion, increasing planting density within a certain range is an effective way to improve seed yield by coordinating the population pod number, seed number per pod, and 1000-seed weight.

### 3.2. Effects of Planting Density on Biomass

Sufficient accumulation and reasonable distribution of biomass is a crucial foundation for achieving a high yield. In this study, increasing planting density within the range of D1 to D3 increased the accumulation of biomass in various organs, particularly in pods and seeds. However, an excessive planting density led to a decline in biomass accumulation, even falling below that in D1. This suggested that optimizing planting density is beneficial for promoting biomass accumulation, which was consistent with previous studies [14]. Rational allocation of biomass facilitated nutrient absorption and transport. In this study, with an increased planting density, there was a gradual decrease in the proportion of biomass in roots and stems, but this increased it in pods. Roots and stems are organs that support structures at the mature stage. Pod plays a pivotal role as a photosynthetic organ to provide substantial carbohydrates for seed filling. These results indicated that rapeseed plants under a high planting density could increase the investment in pods to promote pod growth and enhanced pod productivity. Furthermore, this study revealed an initial increase followed by a subsequent decrease trend in the proportion of biomass in seeds with increasing planting density, which suggested that increasing planting density within a certain range promoted nutrient transfer towards seeds and improved the harvest index.

### 3.3. Effects of Planting Density on Canopy Structure

An optimized canopy structure is beneficial to the efficient utilization of nutrient and light resources, thereby promoting seed filling and increasing the crop yield [35,36]. The microenvironment varies with different positions within the canopy, which consequently leads to variation in the seed yield [37]. Hence, we classified pods based on their positions in main raceme and branch raceme to further investigate the influence of the canopy structure on the seed yield. The pod number, number of seeds per pod, 1000-seed weight, and seed yield in main raceme were significantly higher than those in branch raceme; thus, the pods in main raceme may be considered high-productivity pods compared to other pods in branch raceme. Taking advantage of the high productivity of pods in main raceme was an effective way to increase seed yield. In our study, the yield distribution of main raceme in D2 and D3 reached about 30%, which was significantly higher than that in D1, resulting in a higher seed yield. Although the yield distribution of main raceme in D4 and D5 was higher than that in D2 and D3, the decreased pod quality (number of seeds per pod and 1000-seed weight) reduced the overall population productivity and consequent seed yield.

The pod is the main organ of photosynthesis during the seed-filling stage, and PAI can reflect the light utilization efficiency. In this study, as the planting density increased, the PAI initially increased and then fell, reaching its highest value at D3 or D4. Wang et al. found that as the planting density increased from 2 × 10^5^ plants ha^−1^ to 6 × 10^5^ plants ha^−1^, there was a considerable increase in PAI; furthermore, as the density continued to increase up to 12 × 10^5^ plants ha^−1^, PAI tended to stabilize [38]. In our experiment, we observed that the D5 treatment resulted in a reduction in the quantity of pods and a drop in individual pod size, which may explain the observed decline in PAI under an excessively high density. By increasing planting density, light interception significantly increased, suggesting a higher planting density could enhance the utilization of light resources. Li et al. also reported that compared to a low planting density of 2.7 ×10^5^ plants ha^−1^, the light interception rate under a high planting density of 5.85 × 10^5^ plants ha^−1^ increased from 74.7% to 93.8% [39]. Moreover, we also found that increasing planting density altered the light distribution in the canopy. As the planting density increased, there was a corresponding increase in the ratio of light distributed in the upper part of the canopy, while there was a decrease in the ratio of light distributed in the lower part. This indicated that a high planting density inhibited the transmission of light through the canopy, leading to an uneven distribution of light within the canopy. The light reaching the low layer of canopy was lower, causing a decrease in the pod photosynthetic rate of the lower branches. From the perspective of seed yield distribution, the number of seeds per pod and 1000-seed weight of lower branches decreased with the increasing planting density because of the uneven light distribution. Therefore, efforts at regulating planting density should consider both the positive effect of enhancing light interception and the adverse effect of deteriorating light distribution.

Overall, D2 and D3 exhibited the optimal proportions between the main raceme and branch raceme, with a ratio of 1:8–9. Approximately 30.0% of the seed yield was produced by the main raceme, while the remaining 70.0% was produced by the branch racemes. This distribution effectively maximized the productivity advantage of the main raceme. Furthermore, the PAI in D2 and D3 exhibited elevated levels ranging from 5.4 to 5.8. The whole canopy effectively intercepted approximately 93% of the light, and the distribution of light was more evenly spread. Specifically, the upper and lower parts of the canopy had a distribution ratio of approximately 7:3. Additionally, the pod photosynthetic rate remained consistently high. Therefore, D2 and D3 produced the highest seed yields.

## 4. Materials and Methods

### 4.1. Experimental Site and Design

This experiment was carried out on the Shiye Experimental Farm (32°23′ N, 119°31′ E), Zhenjiang, Jiangsu Province, China, during the 2021–2022 and 2022–2023 rapeseed growing seasons. The rainfall and mean temperature during the two growing seasons are presented in Table 7. The soil texture (0–20 cm) was sandy loam, with organic matter 23.2 g kg^−1^, available nitrogen 117.5 mg kg^−1^, available phosphorus 24.5 mg kg^−1^, and available potassium 92.6 mg kg^−1^. The experiment was conducted with a randomized block design with three replicates. The planting density levels were set as D1 (2.4 × 10^5^ plants ha^−1^), D2 (3.6 × 10^5^ plants ha^−1^), D3 (5.4 × 10^5^ plants ha^−1^), D4 (6.0 × 10^5^ plants ha^−1^), and D5 (7.2 × 10^5^ plants ha^−1^), respectively. Hybrid varieties of Qinyou10 and Ningza1838 were used in this experiment. The plot size was 20 m × 2.4 m (length × width). The seeds were sown on 10 October 2021 and 11 October 2022. The row spacing in all plots was set at 40 cm. The seedling density was adjusted to a relative density level by regulating plant spacing (10.4 cm for D1, 6.9 cm for D2, 5.2 cm for D3, 4.2 cm for D2, and 3.5 cm for D5) at the fourth- and fifth-leaf stages. Compound fertilizer (N-P-K, 15%-15%-15%) and boron fertilizer (B, 12%) were applied at the rates of 900 kg ha^−1^ and 4.5 kg ha^−1^ as basal fertilizers. Urea was applied at the rate of 117.4 kg ha^−1^ as a seedling fertilizer and 176.1 kg ha^−1^ as a bolting fertilizer. Thus, the total application rates of N, P, and K were 270 kg N ha^−1^, 135 kg P_2_O_5_ ha^−1^, and 135 kg K_2_O ha^−1^, respectively.

### 4.2. Sampling and Measurement

#### 4.2.1. Plot Seed Yield and Seedling Survival Rate

At the mature stage, plants of 14.4 m^2^ (6 length × 2.4 width) in each plot were manually harvested, then air-dried for 5–6 days and threshed to measure the seed yield. Then, the harvested density was counted; the seedling survival rate was calculated by dividing the planting density by the harvested density.

#### 4.2.2. Yield Components

At the mature stage, 6 plants for D1, 9 plants for D2, 12 plants for D3, 15 plants for D4, and 18 plants for D5 were sampled. The pods were divided based on the position of the branch raceme and main raceme; then, the number of pods, 1000-seed weight, and seed yield of each position were counted or measured; and the number of pods per pod was calculated by dividing the seed yield by the number of pods and 1000-seed weight. Following that, the overall yield components were calculated according to the above data.

#### 4.2.3. Biomass Accumulation and Partitioning

At the mature stage, the above-mentioned plants for measuring yield components were also used to measure biomass accumulation and partitioning. These plants were divided into the root, stem, pod, and seed, then dried at 105 °C for 30 min, followed by 85 °C to reach a constant weight. The biomass partitioning into different organs was calculated by dividing the biomass in that organ by the total biomass.

#### 4.2.4. PAI and Pod Photosynthesis

At the seed-filling stage, 5 successive plants in each plot were sampled to measure the length and width of each pod by using Clarke’s formula: Sa = πdh^1^ + 1/3πdh^2^, where h^1^ = 0.8 H; h^2^ = 0.2 H; H is the length of the pod; and d is the width of the pod. The PAI was calculated using the measured area.

Three representative plants in each plot were selected at the seed-filling stage. The pods were categorized into three groups based on their positions: main raceme, middle branch raceme, and low branch raceme. For example, if a plant had seven branches, the measurement would be taken for the pods located in the main raceme, the raceme of the fourth branch, and the raceme of the lowest branch. Three green pods in a raceme were selected to measure the photosynthetic rate using a convenient photosynthesis tester (LI-6400xt, LI-COR, Nebraska, NE, USA). The measurement was conducted during the period of 9:00–11:00 on a sunny day with the chamber light intensity set at 1200 μmol m^−2^ s^−1^, the CO_2_ concentration at 400 μmol mol^−1^, and the temperature at 25 °C, respectively.

#### 4.2.5. Light Interception and Distribution

At the seed-filling stage, three sites in each plot were selected to measure light interception and distribution. The instantaneous photosynthetically active radiation (IAPR) was measured at the top of the canopy, the middle layer of the canopy, and the bottom of the canopy using a canopy analyzer (AccuPAR LP-80, Meter, Washinton, WA, USA). For instance, if the thickness (the distance from the lowest pod to the highest pod) of the canopy in a plant population was 100 cm, the measurements would be taken at the top of the canopy, the 50 cm height of the canopy, and the bottom of the canopy.

The light interception rate (LI) and light distribution ratio (LDR) were calculated as follows:

$$LI\;of\;the\;upper\;part\;of\;canopy=\frac{IPAR\;of\;the\;middle\;layer\;of\;canopy}{IPAR\;of\;the\;top\;of\;canopy}$$

$$LI\;of\;the\;whole\;canopy=\frac{IPAR\;of\;the\;bottom\;of\;canopy}{IPAR\;of\;the\;top\;of\;canopy}$$

$$LDR\;of\;the\;upper\;part\;of\;canopy=\frac{LI\;of\;the\;upper\;part\;of\;canopy}{LI\;of\;the\;whole\;canopy}$$

$$LDR\;of\;the\;lower\;part\;of\;canopy=\frac{LI\;of\;the\;whole\;canopy-LI\;of\;the\;upper\;part\;of\;canopy}{LI\;of\;the\;whole\;canopy}$$

### 4.3. Statistical Analysis

The analysis of variance (ANOVA) was performed with SPSS 20 software (SPSS Inc., Chicago, IL, USA), and means were compared by the least significant difference test (LSD) at the *p* = 0.05 level. Graphs were prepared using Origin 9.0 software (OriginLab Corp, Massachusetts, MA, USA).

## 5. Conclusions

Under D2 and D3, a greater number of pods in the population, number of seeds per plant, and 1000-seed weight were produced; therefore, the seed yield under these densities was the highest. When further increasing planting density to D4 and D5, the intensity of individual competition led to a decrease in seed yield. Increasing the planting density from D1 to D3 enhanced biomass accumulation and biomass partitioning in seeds, indicating greater productivity and a higher harvest index. In addition, D2 and D3 exhibited the optimal proportions between the main raceme and branch raceme, with a ratio of 1:8–9. The D2 and D3 treatments produced high levels of PAI and light interception, along with an even light distribution within the canopy. Therefore, the planting density of 3.6–5.4 × 10^5^ plants ha^−1^ is recommended in rapeseed production.

## Figures and Tables

**Table 1:** Effects of planting density on yield components of canola plants.

Year	Variety	Planting Density	Seedling Survival Rate (%)	Yield per Plant (g)	Number of Pods per Plant	Number of Pods in Population (×10^6^ ha^−1^)	Number of Seeds per Pod	1000-Seed Weight (g)
2021–2022	Qinyou10	D1	97.8 a	19.48 a	333.0 a	78.16 d	17.5 a	3.502 b
		D2	97.2 a	14.50 b	243.4 b	85.15 c	17.8 a	3.626 a
		D3	96.7 a	11.24 c	203.0 c	94.23 a	16.2 b	3.663 a
		D4	93.6 b	7.76 d	170.8 d	95.92 a	15.6 c	3.182 c
		D5	91.6 b	5.86 e	138.4 e	91.26 b	15.1 d	3.099 d
	Ningza1838	D1	97.4 a	19.55 a	333.2 a	77.89 d	17.6 a	3.674 a
		D2	97.1 a	15.18 b	236.6 b	82.67 c	17.7 a	3.750 a
		D3	95.9 a	11.44 c	195.8 c	90.13 b	16.1 b	3.766 a
		D4	94.5 ab	7.89 d	167.4 d	94.86 a	15.1 c	3.313 b
		D5	92.0 b	5.93 e	140.3 e	92.98 ab	14.9 c	3.114 c
2022–2023	Qinyou10	D1	97.9 a	18.61 a	331.9 a	77.96 d	17.6 a	3.466 b
		D2	97.1 a	13.95 b	244.0 b	85.30 c	17.5 a	3.599 a
		D3	94.8 b	10.97 c	204.4 c	92.96 a	16.2 b	3.614 a
		D4	92.0 c	7.66 d	170.5 d	94.13 a	15.5 c	3.128 c
		D5	89.5 d	5.74 e	136.9 e	88.18 b	14.9 d	3.096 c
	Ningza1838	D1	97.9 a	19.19 a	328.3 a	77.11 d	17.3 a	3.620 b
		D2	96.5 b	14.84 b	239.1 b	83.02 c	17.6 a	3.777 a
		D3	95.4 c	11.04 c	196.5 c	89.96 b	16.1 b	3.777 a
		D4	92.3 d	7.73 d	167.4 d	92.64 a	15.1 c	3.293 c
		D5	89.0 e	5.93 e	138.1 e	88.48 b	14.9 c	3.172 d
ANOVA								
Year			**	**	ns	**	ns	ns
Variety			ns	**	**	**	*	**
Planting density			**	**	**	**	**	**
Year × variety			ns	ns	ns	ns	ns	ns
Year × planting density			ns	ns	ns	*	ns	ns
Variety × planting density			ns	ns	**	**	ns	*
Year × variety × planting density			ns	ns	ns	ns	ns	ns

Different letters indicate significant difference at *p* = 0.05 for the same year and same variety between different planting density levels. D1, 2.4 × 10^5^ plants ha^−1^; D2, 3.6 × 10^5^ plants ha^−1^; D3, 5.4 × 10^5^ plants ha^−1^; D4, 6.0 × 10^5^ plants ha^−1^; D5, 7.2 × 10^5^ plants ha^−1^. Probability levels are represented by ns, *, and ** for not significant, 0.05, and 0.01.

**Table 2:** Effects of planting density on biomass accumulation (kg ha^−1^) of canola plants.

Year	Variety	Planting Density	Root	Stem	Pod	Seed	Total Plant
2021–2022	Qinyou10	D1	1772.7 b	8021.2 b	4823.7 bc	4785.9 b	19,403.4 b
		D2	1856.9 ab	8678.7 a	5367.3 a	5495.7 a	21,398.6 a
		D3	1882.3 a	8832.5 a	5553.8 a	5606.4 a	21,875.0 a
		D4	1580.4 c	7631.8 b	4976.6 b	4764.3 b	18,953.0 b
		D5	1412.0 d	6868.7 c	4563.9 c	4264.2 c	17,108.8 c
	Ningza1838	D1	1835.6 a	8040.5 b	5123.4 b	5022.6 b	20,022.1 b
		D2	1852.5 a	8447.8 ab	5331.7 ab	5489.0 a	21,121.0 a
		D3	1854.0 a	8512.6 a	5544.2 a	5469.8 a	21,380.6 a
		D4	1589.9 b	7490.6 c	5127.1 b	4746.7 c	18,954.3 c
		D5	1430.5 c	6874.1 d	4702.1 c	4315.2 d	17,321.8 d
2022–2023	Qinyou10	D1	1748.0 b	7922.4 b	4739.8 c	4756.3 b	19,166.5 c
		D2	1828.4 a	8480.4 a	5156.8 b	5379.5 a	20,845.0 b
		D3	1834.9 a	8612.2 a	5497.8 a	5454.4 a	21,399.3 a
		D4	1513.9 c	7330.8 c	4793.9 c	4553.7 c	18,192.4 d
		D5	1351.3 d	6537.4 d	4354.1 d	4079.8 d	16,322.7 e
	Ningza1838	D1	1771.8 b	7735.1 b	4905.9 b	4842.9 b	19,255.7 b
		D2	1860.2 a	8575.9 a	5423.9 a	5529.3 a	21,389.4 a
		D3	1853.3 a	8427.3 a	5460.3 a	5462.2 a	21,203.1 a
		D4	1534.5 c	7382.9 b	5011.4 b	4610.9 c	18,539.8 c
		D5	1381.0 d	6630.2 c	4404.8 c	4188.4 d	16,604.3 d

Different letters indicate significant difference at *p* = 0.05 for the same year and same variety between different planting density levels. D1, 2.4 × 10^5^ plants ha^−1^; D2, 3.6 × 10^5^ plants ha^−1^; D3, 5.4 × 10^5^ plants ha^−1^; D4, 6.0 × 10^5^ plants ha^−1^; D5, 7.2 × 10^5^ plants ha^−1^.

**Table 3:** Effects of planting density on biomass partitioning (%) of canola plants.

Year	Variety	Planting Density	Root	Stem	Pod	Seed
2021–2022	Qinyou10	D1	9.14 a	41.33 a	24.85 c	24.68 b
		D2	8.68 b	40.55 ab	25.09 c	25.68 a
		D3	8.61 bc	40.38 ab	25.39 bc	25.63 a
		D4	8.34 cd	40.27 b	26.25 ab	25.14 ab
		D5	8.25 d	40.15 b	26.68 a	24.93 b
	Ningza1838	D1	9.17 a	40.16 a	25.59 b	25.08 bc
		D2	8.77 b	40.00 a	25.24 b	25.99 a
		D3	8.67 b	39.81 a	25.93 b	25.59 ab
		D4	8.39 c	39.52 a	27.04 a	25.05 c
		D5	8.26 c	39.68 a	27.15 a	24.91 c
2022–2023	Qinyou10	D1	9.12 a	41.33 a	24.73 c	24.82 b
		D2	8.77 b	40.68 ab	24.74 c	25.81 a
		D3	8.57 bc	40.24 b	25.70 b	25.49 ab
		D4	8.32 c	40.29 b	26.35 ab	25.03 b
		D5	8.28 c	40.05 b	26.68 a	24.99 b
	Ningza1838	D1	9.20 a	40.18 a	25.48 c	25.15 c
		D2	8.70 b	40.08 a	25.37 c	25.85 a
		D3	8.74 b	39.74 a	25.75 bc	25.76 ab
		D4	8.28 c	39.82 a	27.03 a	24.87 c
		D5	8.32 c	39.93 a	26.53 ab	25.22 bc

Different letters indicate significant difference at *p* = 0.05 for the same year and same variety between different planting density levels. D1, 2.4 × 10^5^ plants ha^−1^; D2, 3.6 × 10^5^ plants ha^−1^; D3, 5.4 × 10^5^ plants ha^−1^; D4, 6.0 × 10^5^ plants ha^−1^; D5, 7.2 × 10^5^ plants ha^−1^.

**Table 4:** Effects of planting density on yield components of main raceme and branch raceme during the 2021–2022 growing season.

Items	Variety	Planting Density	Main Stem	Branch Racemes from Top to Bottom									
				1	2	3	4	5	6	7	8	9	10
Pod number in population (×10^6^ ha^−1^)	Qinyou 10	D1	16.48	5.46	6.27	7.32	7.49	7.42	7.14	6.84	5.92	4.70	3.15
		D2	21.74	5.97	7.73	8.40	8.51	8.98	7.78	6.56	5.84	3.66	
		D3	25.93	7.43	7.66	8.52	9.59	10.19	8.94	8.40	7.63		
		D4	28.79	9.02	8.76	10.78	11.43	11.12	9.56	6.64			
		D5	33.27	6.30	8.02	11.89	12.66	8.69	10.62				
	Ningza 1838	D1	16.59	4.88	6.09	7.40	7.28	7.39	6.73	6.86	4.87	4.54	5.31
		D2	21.37	5.83	7.84	7.89	8.44	8.59	7.64	6.27	5.27	3.56	
		D3	25.64	7.24	7.26	8.18	9.03	9.77	7.89	8.04	7.22		
		D4	28.79	8.78	8.39	10.36	11.52	9.98	8.93	8.26			
		D5	32.37	6.33	8.00	10.67	12.62	12.03	11.18				
Seed number per pod	Qinyou 10	D1	18.0	15.8	17.1	16.7	18.1	18.2	18.6	18.5	16.6	17.1	14.8
		D2	18.7	16.4	17.6	17.3	18.7	17.7	19.2	18.3	16.2	13.0	
		D3	16.9	17.2	16.4	16.1	15.2	15.4	16.9	14.9	16.4		
		D4	18.3	10.3	15.4	16.4	13.5	17.5	17.0	8.6			
		D5	15.9	14.2	10.9	14.3	16.1	16.1	14.8				
	Ningza 1838	D1	18.2	14.9	16.1	16.7	18.2	18.6	16.7	20.9	17.6	17.3	15.4
		D2	18.9	17.1	16.9	17.6	18.0	18.5	18.6	16.9	15.5	14.3	
		D3	17.0	17.8	14.3	15.4	15.3	17.4	15.5	14.4	16.0		
		D4	16.6	14.3	14.6	15.6	15.8	15.2	15.6	9.2			
		D5	15.8	12.5	14.0	14.6	15.7	14.7	13.8				
1000-seed weight (g)	Qinyou 10	D1	4.012	3.595	3.599	3.369	3.536	3.285	3.376	3.222	3.161	3.275	2.994
		D2	3.833	3.576	3.617	3.825	3.441	3.585	3.524	3.309	3.468	3.578	
		D3	3.893	3.626	3.745	3.741	3.671	3.593	3.378	3.517	3.304		
		D4	3.548	3.145	3.247	2.927	2.968	2.988	2.796	2.852			
		D5	3.360	3.059	2.997	2.991	2.951	2.803	2.887				
	Ningza 1838	D1	4.089	3.909	3.780	3.594	3.630	3.597	3.571	3.478	3.343	3.487	3.136
		D2	4.084	3.835	3.661	3.875	3.484	3.643	3.549	3.471	3.568	3.346	
		D3	4.155	3.662	3.835	3.662	3.599	3.637	3.447	3.567	3.397		
		D4	3.529	3.384	3.412	3.226	3.247	3.183	3.007	2.871			
		D5	3.284	3.221	2.947	3.064	2.994	2.947	3.010				

D1, 2.4 × 10^5^ plants ha^−1^; D2, 3.6 × 10^5^ plants ha^−1^; D3, 5.4 × 10^5^ plants ha^−1^; D4, 6.0 × 10^5^ plants ha^−1^; D5, 7.2 × 10^5^ plants ha^−1^.

**Table 5:** Effects of planting density on yield components of main raceme and branch raceme during the 2022–2023 growing season.

Items	Variety	Planting Density	Main Stem	Branch Racemes from Top to Bottom									
				1	2	3	4	5	6	7	8	9	10
Pod number in population (×10^6^ ha^−1^)	Qinyou 10	D1	15.98	5.51	6.24	7.41	7.41	7.52	7.15	6.84	6.05	4.71	3.15
		D2	21.27	6.02	7.98	8.36	8.67	8.97	7.85	6.57	5.91	3.70	
		D3	25.09	7.42	7.44	8.65	9.44	10.07	8.91	8.35	7.59		
		D4	27.40	8.91	8.46	10.66	11.52	11.11	9.39	6.68			
		D5	31.14	6.22	7.65	11.53	12.48	8.64	10.53				
	Ningza 1838	D1	16.15	4.88	6.10	7.27	7.27	7.39	6.62	6.72	4.87	4.58	5.27
		D2	21.04	5.83	7.80	8.16	8.47	8.78	7.67	6.39	5.36	3.52	
		D3	24.81	7.19	7.23	8.20	9.21	9.84	7.99	8.12	7.37		
		D4	27.60	8.64	8.18	10.37	11.24	9.69	8.82	8.09			
		D5	29.79	6.16	7.70	10.26	12.11	11.70	10.77				
Seed number per pod	Qinyou 10	D1	17.9	15.9	17.4	16.9	18.3	18.6	18.8	18.6	16.8	17.0	15.0
		D2	18.5	16.1	17.3	17.1	18.5	17.6	18.8	17.9	16.0	12.5	
		D3	17.1	16.8	16.3	16.1	15.3	15.4	16.9	14.9	15.9		
		D4	18.1	10.5	15.3	16.5	13.4	17.5	16.7	8.5			
		D5	16.1	13.7	10.6	14.2	15.8	15.9	14.5				
	Ningza 1838	D1	17.8	14.8	16.1	16.6	18.0	18.7	16.6	20.7	17.1	17.4	15.0
		D2	18.9	16.6	16.8	17.2	18.5	18.1	18.7	16.7	15.8	14.0	
		D3	16.8	17.7	14.6	15.1	15.3	17.3	15.7	14.5	16.2		
		D4	16.5	14.1	15.0	15.4	15.7	15.4	16.1	9.4			
		D5	15.9	12.8	14.0	14.5	15.9	14.6	13.9				
1000-seed weight (g)	Qinyou 10	D1	3.945	3.578	3.518	3.382	3.501	3.265	3.303	3.261	3.166	3.252	2.999
		D2	3.854	3.543	3.619	3.782	3.360	3.523	3.512	3.319	3.378	3.479	
		D3	3.861	3.576	3.634	3.654	3.589	3.515	3.367	3.422	3.384		
		D4	3.478	3.139	3.238	2.927	2.912	2.918	2.724	2.839			
		D5	3.346	3.101	2.977	2.972	2.964	2.839	2.887				
	Ningza 1838	D1	4.085	3.807	3.708	3.507	3.549	3.572	3.531	3.430	3.310	3.379	3.054
		D2	4.106	3.803	3.742	3.935	3.582	3.624	3.521	3.540	3.612	3.362	
		D3	4.190	3.666	3.791	3.620	3.688	3.670	3.464	3.514	3.442		
		D4	3.526	3.399	3.396	3.177	3.202	3.133	3.015	2.863			
		D5	3.368	3.315	3.048	3.083	3.033	2.981	3.050				

D1, 2.4 × 10^5^ plants ha^−1^; D2, 3.6 × 10^5^ plants ha^−1^; D3, 5.4 × 10^5^ plants ha^−1^; D4, 6.0 × 10^5^ plants ha^−1^; D5, 7.2 × 10^5^ plants ha^−1^.

**Table 6:** Effects of planting density on yield distribution (%) in main raceme and branch raceme.

Year	Variety	Planting Density	Main Raceme	Branch Raceme from Top to Bottom									
				1	2	3	4	5	6	7	8	9	10
2021– 2022	Qinyou10	D1	24.83	6.46	8.01	8.61	10.01	9.26	9.38	8.54	6.50	5.48	2.92
		D2	28.41	6.37	8.94	10.08	9.94	10.39	9.57	7.24	5.97	3.08	
		D3	30.46	8.24	8.26	9.15	9.54	10.06	9.09	7.85	7.35		
		D4	39.25	6.13	8.96	10.87	9.63	12.23	9.51	3.42			
		D5	41.69	6.39	6.01	11.94	14.10	12.23	10.67				
	Ningza1838	D1	24.64	5.65	7.32	8.81	9.55	9.87	7.99	9.93	5.7	5.45	5.09
		D2	30.01	6.97	8.81	9.81	9.64	10.53	9.18	6.67	5.30	3.09	
		D3	33.03	8.60	7.15	8.42	9.07	11.32	7.70	7.54	7.18		
		D4	35.40	8.98	8.65	10.97	12.44	10.16	8.81	4.60			
		D5	38.92	5.92	7.46	11.04	13.79	12.07	10.80				
2022– 2023	Qinyou10	D1	23.71	6.60	8.00	8.92	9.96	9.56	9.32	8.71	6.75	5.46	3.00
		D2	28.13	6.38	9.28	10.05	9.99	10.32	9.66	7.26	5.94	2.98	
		D3	30.40	8.17	8.08	9.33	9.49	9.96	9.31	7.78	7.48		
		D4	37.84	6.42	9.21	11.32	9.87	12.44	9.36	3.54			
		D5	41.02	6.47	5.92	11.91	14.34	9.52	10.82				
	Ningza1838	D1	24.25	5.66	7.52	8.69	9.58	10.18	8.01	9.85	5.70	5.56	4.99
		D2	29.47	6.64	8.84	10.01	10.16	10.43	9.10	6.81	5.54	3.00	
		D3	32.00	8.54	7.33	8.20	9.49	11.42	7.93	7.54	7.54		
		D4	34.69	8.96	9.04	10.93	12.29	10.10	9.29	4.70			
		D5	37.99	6.24	7.83	10.98	13.94	12.11	10.91				

D1, 2.4 × 10^5^ plants ha^−1^; D2, 3.6 × 10^5^ plants ha^−1^; D3, 5.4 × 10^5^ plants ha^−1^; D4, 6.0 × 10^5^ plants ha^−1^; D5, 7.2 × 10^5^ plants ha^−1^.

**Table 7:** Rainfall and mean temperature during rapeseed growing season in 2021–2023.

Year	Items	October	November	December	January	February	March	April	May
2021–2022	Rainfall (mm)	98.0	36.6	5.4	77.1	25.3	166.1	86.2	15.5
	Mean temperature (°C)	18.8	11.6	5.0	3.9	4.0	12.6	17.3	21.3
2022–2023	Rainfall (mm)	131.6	74.0	8.9	40.9	69.0	35.5	43.1	72.5
	Mean temperature (°C)	16.7	13.7	2.8	3.7	5.5	12.8	17.0	21.8

## Data Availability

Data will be made available on request.
